# Presence of the Herbaceous Marsh Species *Schoenoplectus americanus* Enhances Surface Elevation Gain in Transitional Coastal Wetland Communities Exposed to Elevated CO_2_ and Sediment Deposition Events

**DOI:** 10.3390/plants11091259

**Published:** 2022-05-06

**Authors:** Camille LaFosse Stagg, Claudia Laurenzano, William C. Vervaeke, Ken W. Krauss, Karen L. McKee

**Affiliations:** 1 U.S. Geological Survey, Wetland and Aquatic Research Center, Lafayette, LA 70506, USA; william_vervaeke@nps.gov (W.C.V.); kraussk@usgs.gov (K.W.K.); mckeek@usgs.gov (K.L.M.); 2 Cherokee Nation System Solutions, Contractor to the U.S. Geological Survey, Wetland and Aquatic Research Center, Lafayette, LA 70506, USA; claurenzano@contractor.usgs.gov

**Keywords:** climate change, coastal wetlands, multiple stressors, elevated CO_2_, hurricanes, sediment deposition, wetland elevation change, marsh encroachment, biomass production, cellulose decomposition, mesocosm experiment

## Abstract

Coastal wetlands are dynamic ecosystems that exist along a landscape continuum that can range from freshwater forested wetlands to tidal marsh to mudflat communities. Climate-driven stressors, such as sea-level rise, can cause shifts among these communities, resulting in changes to ecological functions and services. While a growing body of research has characterized the landscape-scale impacts of individual climate-driven stressors, little is known about how multiple stressors and their potential interactions will affect ecological functioning of these ecosystems. How will coastal wetlands respond to discrete climate disturbances, such as hurricane sediment deposition events, under future conditions of elevated atmospheric CO_2_? Will these responses vary among the different wetland communities? We conducted experimental greenhouse manipulations to simulate sediment deposition from a land-falling hurricane under future elevated atmospheric CO_2_ concentrations (720 ppm CO_2_). We measured responses of net primary production, decomposition, and elevation change in mesocosms representing four communities along a coastal wetland landscape gradient: freshwater forested wetland, forest/marsh mix, marsh, and mudflat. When *Schoenoplectus americanus* was present, above- and belowground biomass production was highest, decomposition rates were lowest, and wetland elevation gain was greatest, regardless of CO_2_ and sediment deposition treatments. Sediment addition initially increased elevation capital in all communities, but post-deposition rates of elevation gain were lower than in mesocosms without added sediment. Together these results indicate that encroachment of oligohaline marshes into freshwater forested wetlands can enhance belowground biomass accumulation and resilience to sea-level rise, and these plant-mediated ecosystem services will be augmented by periodic sediment pulses from storms and restoration efforts.

## 1. Introduction

Coastal wetlands are valuable ecosystems that enhance coastal resilience [1,2] and reduce greenhouse gas emissions through carbon sequestration [3,4,5]. Because coastal wetlands provide these and numerous other critical services [6,7], it is imperative to understand how these ecosystems will function under future climate conditions. Existing at the terrestrial–aquatic interface, coastal wetlands are naturally resilient ecosystems that have been sculpted by dynamic climate and flooding conditions for millennia [8,9]. Low-lying coastal wetlands must keep pace with changes in sea level or become submerged. Wetlands adjust to rising sea levels through a positive hydrogeomorphic feedback mechanism that achieves optimal wetland elevation and flooding through processes that control mineral sediment and organic matter accumulation [10,11,12], leading to enhanced coastal habitat stability and soil carbon sequestration [13,14,15]. However, excessive flooding that surpasses a critical elevation threshold can disrupt the hydrogeomorphic feedback, leading to an ecosystem shift or collapse [16,17,18].

**Figure 1:**
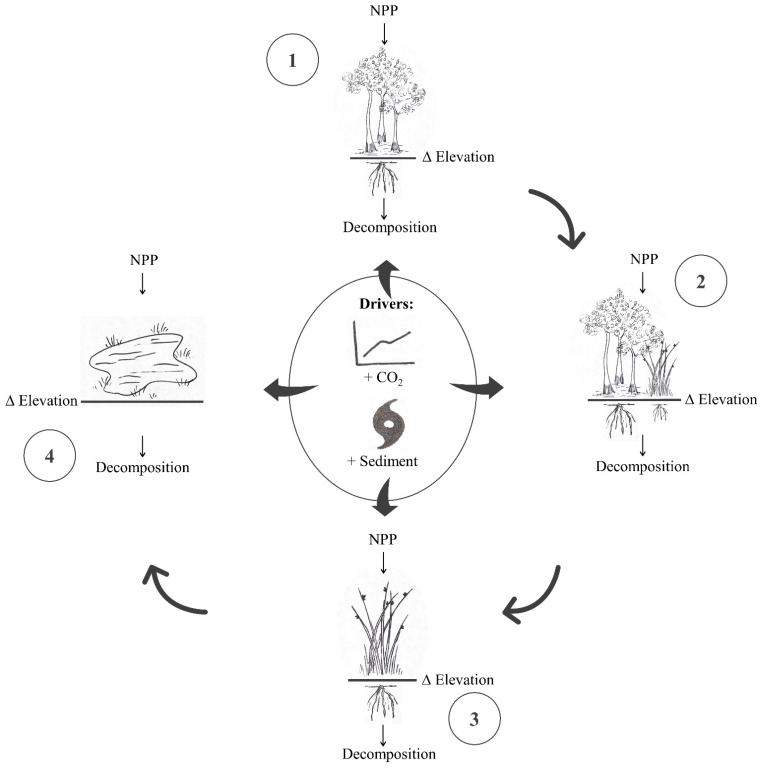
Conceptual experimental design. Organic matter inputs through net primary production (NPP) counter-balance organic matter exports through decomposition resulting in net elevation surplus or deficit in four wetland communities along a landscape-scale transition (clockwise) from (1) freshwater forested wetland to (2) transitional mixed forest/marsh, to (3) marsh, to (4) mudflat. Experimental manipulations (elevated CO_2_ and sediment deposition) simulated future hurricane sediment deposition events in a CO_2_-enriched environment.

Coastal freshwater forested wetlands occur at the upper limit of the tidal range and are vulnerable to hydrologic alterations, including saltwater intrusion [19]. Sea-level rise can cause shifts in plant community composition along a continuum ranging from freshwater forested wetland to unvegetated mudflat [20], potentially altering ecosystem function [21,22]. Conservation and management of these low-lying ecosystems requires an understanding of how future climate conditions can affect wetland resilience to sea-level rise and how it may vary along this coastal wetland landscape continuum (Figure 1).

The most recent IPCC report (IPCC AR6) makes it clear that increasing greenhouse gas emissions, including atmospheric CO_2_, will cause faster warming with cascading impacts across the globe [23]. In addition to driving global warming, elevated atmospheric CO_2_ concentrations can directly impact coastal wetland ecosystem function. For example, enriched atmospheric CO_2_ can stimulate primary production and biomass contributions to soil volume, contributing to elevation gains [24,25]. Effects of elevated atmospheric CO_2_ on decomposition in coastal wetlands are less clear [26,27,28] but have the potential to modify elevation change dynamics by altering the net balance of organic matter gains and losses (Figure 1).

Global warming will accelerate sea-level rise, causing more frequent and severe coastal flooding and erosion [23]. Climate extremes are also predicted to increase, with the possibility of larger, more intense hurricanes making landfall in coastal regions [29]. While extreme precipitation associated with more intense hurricanes can have devastating impacts on coastal wetlands [30,31], these ecosystems have adapted to regular storm events, which play a critical role in ecosystem function [32]. For example, hurricanes deposit significant amounts of sediment through storm surge [33,34,35], which can provide immediate elevation gains (elevation capital) and stimulate processes that control wetland elevation gain [36,37].

Although the impacts of individual drivers, such as atmospheric CO_2_ and sediment deposition, are becoming clearer, less is known about the interactive effects of these factors on coastal wetland elevation change. Therefore, the complexity of possible climate futures requires that we investigate multiple drivers together, rather than in isolation, if we hope to have insight into the future of coastal wetlands in a changing environment. The overall aim of this study was to gain a better understanding of how sediment inputs from hurricanes might affect coastal wetland resilience in a future with elevated atmospheric CO_2_. Will sediment addition and CO_2_ enrichment separately or interactively affect plant production–decomposition processes and biogenic contributions to wetland elevation change? Does sediment addition alter post-storm elevation trajectories by stimulating belowground root production or microbial decay? Do elevation responses differ depending on vegetation type? To answer these questions, we conducted a greenhouse mesocosm study to examine responses of *Nyssa biflora* (swamp tupelo) and *Schoenoplectus americanus* (American bulrush) to simulated hurricane sedimentation under current and future atmospheric CO_2_ concentrations.

## 2. Results

**Figure 2:**
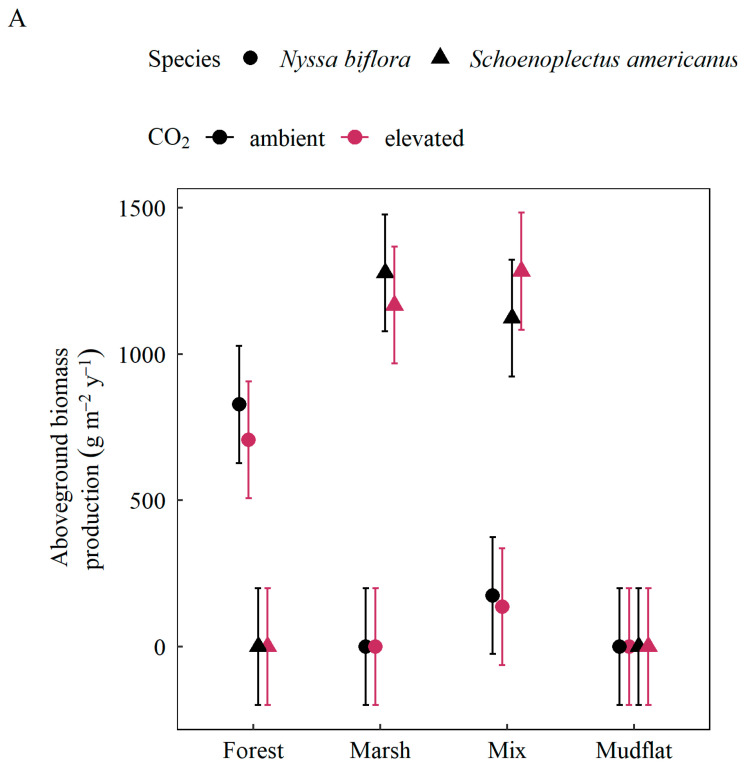

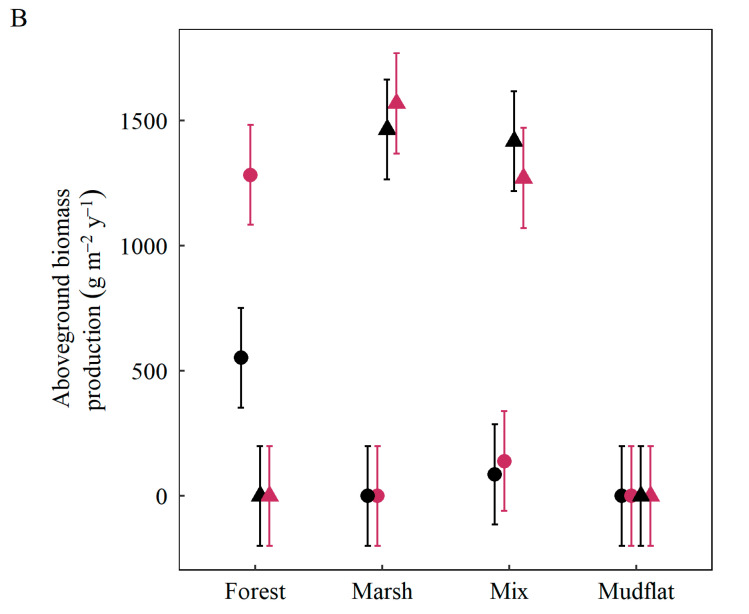
Annual aboveground biomass production of *Nyssa biflora* (circles) and *Schoenoplectus americanus* (triangles) by community and CO_2_ (ambient: black, elevated: magenta). (**A**) No sediment deposition. (**B**) Sediment deposition. Response variable values are model-based estimated marginal means using Satterthwaite approximation. Brackets represent 95% confidence intervals, *p* = 0.052 for full interaction: Species × Community × CO_2_ × Sediment.

Aboveground biomass production was generally highest in the mixed and marsh communities, and high production rates in the mixed community were dominated by the marsh species *Schoenoplectus americanus* (Table 1, Figure 2). Production of the forest species *Nyssa biflora* was lower in the mixed community with *S. americanus* than when grown alone in the forest mesocosms. The addition of elevated CO_2_ and sediment deposition treatments had no effect on this pattern, with the exception of the forested community, which was stimulated by these treatments (*p* = 0.052; Figure 2B). With both elevated CO_2_ and sediment deposition treatments together, *N. biflora* biomass production in the forest mesocosms was similar to *S. americanus* production in the mixed and marsh communities.

**Figure 3:**
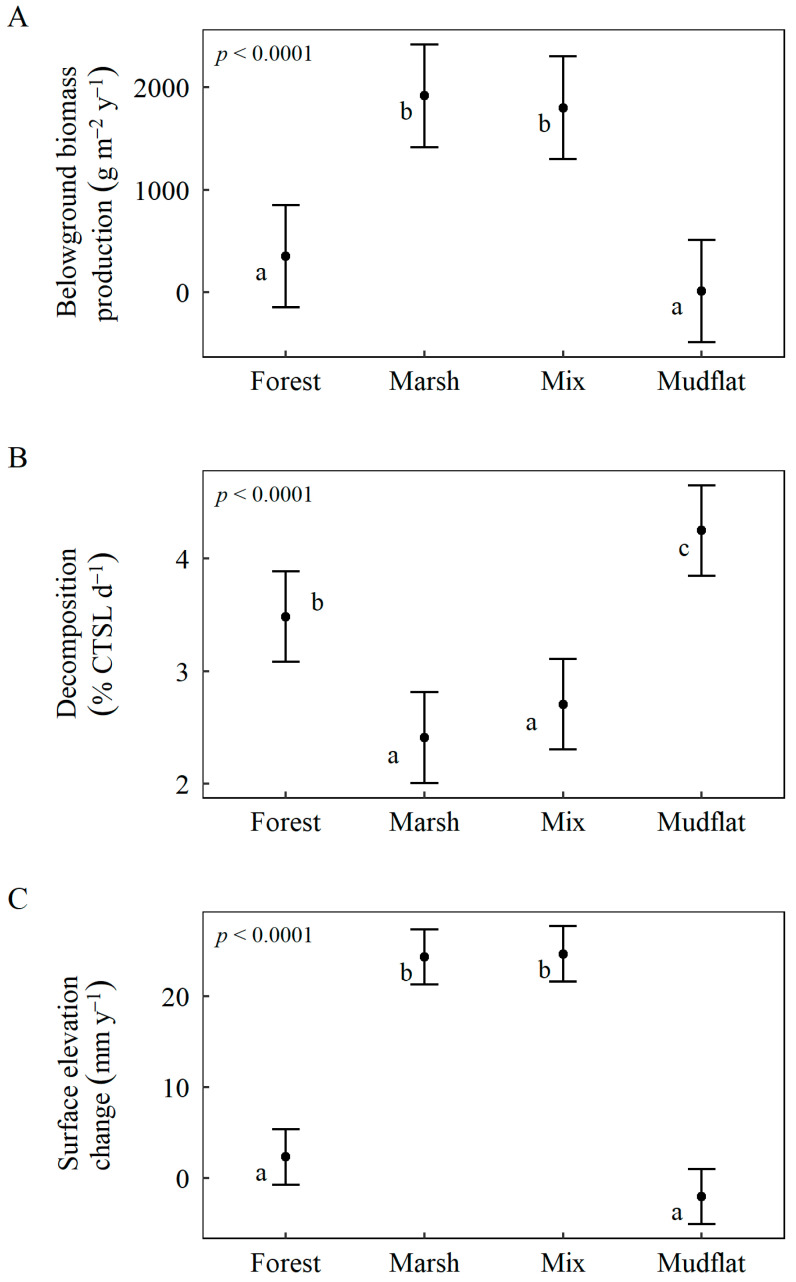
Variation in response variables: (**A**) belowground biomass production, (**B**) decomposition, (**C**) surface elevation change rates among communities. Response variable values are model-based estimated marginal means using Satterthwaite approximation. Brackets represent 95% confidence intervals; letters represent significant differences determined by Tukey’s (*p* < 0.05) post hoc comparisons.

Generally, among all four communities (averaged across CO_2_ and sediment treatments), the marsh and mixed communities had the highest belowground biomass production rates, lowest decomposition rates, and highest surface elevation change rates compared to the forest and mudflat communities (Table 2, Figure 3). While some CO_2_ and sediment treatment effects were dependent upon wetland community, there were no significant interactions between elevated CO_2_ and sediment deposition on belowground production, decomposition, or surface elevation change (Table 2).

**Figure 4:**
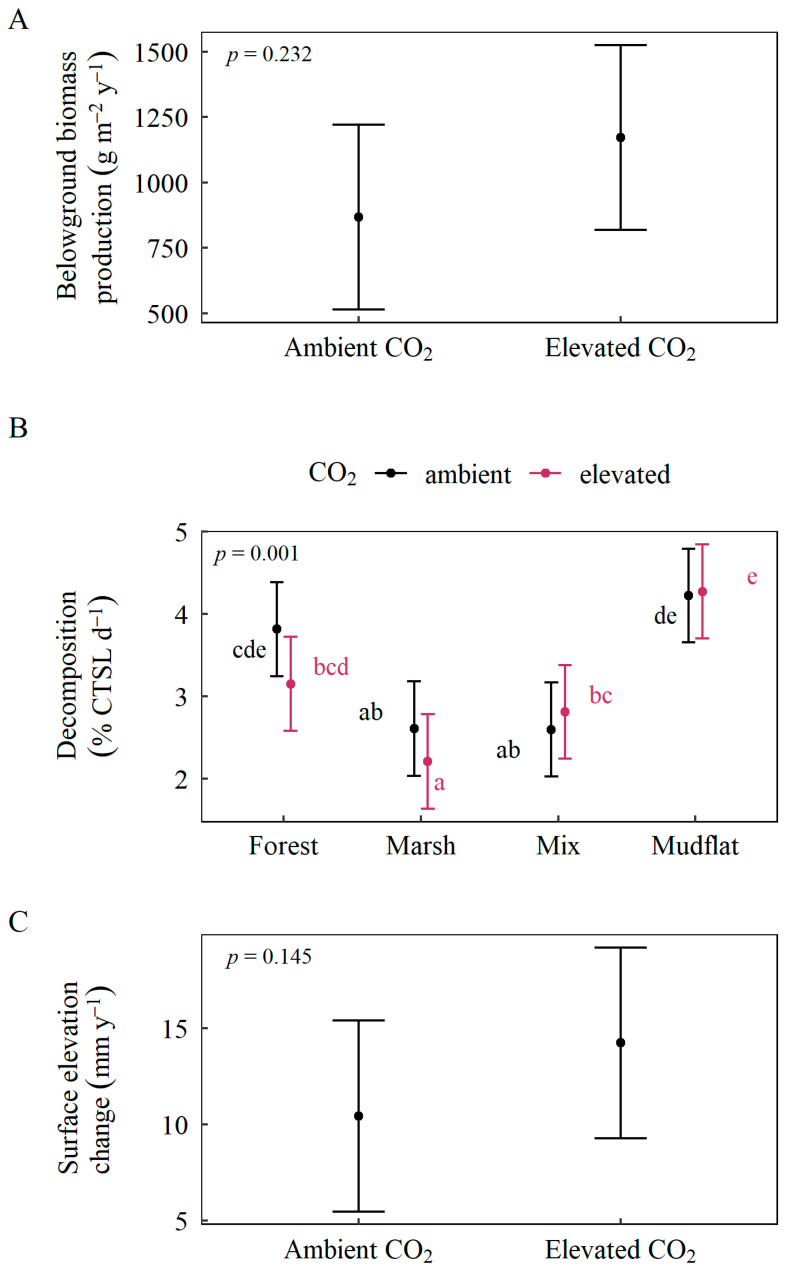
Effects of elevated atmospheric CO_2_ and community interactions, where significant, on all response variables: (**A**) belowground biomass production, (**B**) decomposition, (**C**) surface elevation change rate. Response variable values are model-based estimated marginal means using Satterthwaite approximation. Brackets represent 95% confidence intervals; letters represent significant differences determined by Tukey’s (*p* < 0.05) post hoc comparisons.

Although elevated CO_2_ did not alter belowground biomass production or surface elevation change (Figure 4A,C), there were significant effects on decomposition in the mixed community (Figure 4B). Under ambient CO_2_ conditions, mixed and marsh communities had similar decay rates, and both communities had lower rates of decay compared to the forest and mudflat communities. Under elevated CO_2_ conditions, however, decay rates in the mixed community were more similar to the forest and mudflat communities, and decay rates in the marsh were significantly lower than any other community.

**Figure 5:**
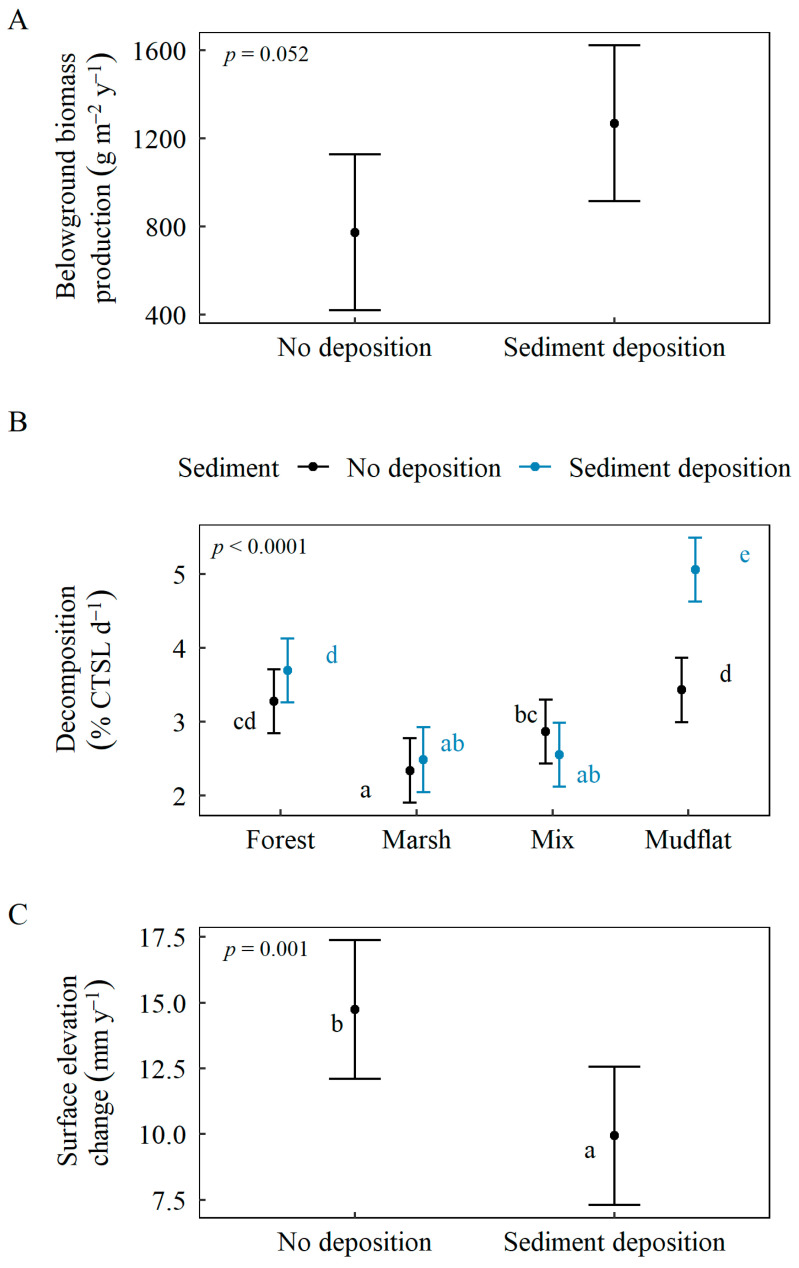
Sediment deposition effects and community interaction, where significant, on all response variables: (**A**) belowground biomass production, (**B**) decomposition, (**C**) surface elevation change rate. Surface elevation change rates represent the period following the sediment deposition event (after day 35, Figure 6B). Response variable values are model-based estimated marginal means using Satterthwaite approximation. Brackets represent 95% confidence intervals; letters represent significant differences determined by Tukey’s (*p* < 0.05) post hoc comparisons.

**Figure 6:**
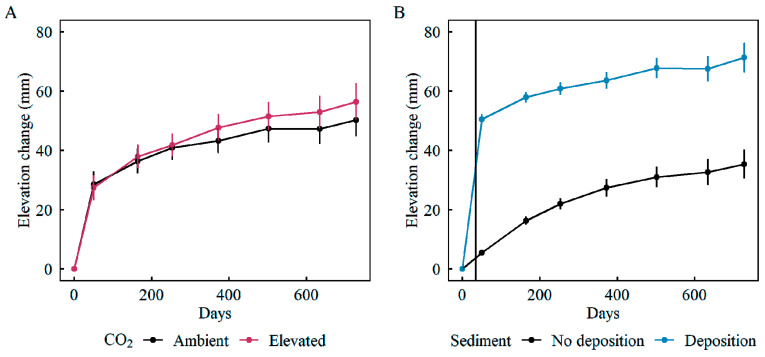
Incremental change in surface elevation in mesocosms over time following (**A**) ambient (black) and elevated (red) CO_2_ treatments and (**B**) no sediment (black) and with sediment (blue) deposition treatments. Data points represent average surface elevation relative to baseline across all communities; brackets represent standard errors. Sediment was added on day 35 (black vertical line).

Sediment deposition had no significant effect on belowground biomass production (Figure 5A). In contrast, sediment deposition stimulated decomposition, but only in the mudflat community (Figure 5B). In all communities, elevation change rates were positive (gaining elevation); however, sediment addition diminished post-deposition rates of wetland elevation gain (Figure 5C). Despite a direct increase in elevation immediately following the deposition of sediment (Figure 6B), the rate of elevation gain in the wetlands receiving sediment was slower than the rate of elevation gain without sediment deposition (Figure 5C and Figure 6B).

## 3. Discussion

Coastal wetlands adjust to rising sea levels through mineral sediment and organic matter accumulation [12,16,38]. In wetlands where vertical land building is dominated by organic matter accumulation, i.e., where mineral sediment is limited [39,40,41,42], changes in the competing processes of organic matter production and decomposition can have significant effects on net wetland elevation change and sustainability [43,44].

Along the coastal wetland landscape continuum, shifts in plant community composition can lead to changes in primary production [45], decomposition [46], and soil organic matter accumulation [47,48]. Following salinity intrusion from sea-level rise, storm events, and drought, the transition from coastal freshwater forested wetland to oligohaline marsh is characterized by declining tree densities, reduced basal area, and lower litterfall rates [49]. In the current study, tree biomass (*N. biflora*) was higher in the forest community compared to tree biomass in the transitional mixed community (*N. biflora* + *S. americanus*). Since salinity was not manipulated in the current study, these results indicate that other factors beyond salinity may affect forest regeneration in these transitional communities. The diminished production of *N. biflora* biomass in the presence of *S. americanus* suggests that *S. americanus*, which had significantly higher biomass production, is a better competitor, with lower resource requirements than *N. biflora* [50,51]. The competitive displacement by *S. americanus* can lead to changes in aboveground production that have implications for carbon cycling. Because woody aboveground biomass in forests is retained over longer periods of time, compared to herbaceous aboveground biomass in marshes, encroachment of *S. americanus* into freshwater forested wetlands could impact the rate of carbon turnover and export from the ecosystem [52,53].

Additionally, mesocosms that contained *S. americanus* (mixed and marsh communities) had the highest rates of belowground production, the lowest decay rates, and the highest rates of surface elevation change. This pattern matches that observed in the field. For example, some tidal freshwater forested wetlands along the Southeastern U.S. Atlantic coasts have lower rates of surface elevation change compared to oligohaline herbaceous marshes closer to the marine tidal source [54]. Herbaceous marshes can also have significantly higher rates of belowground biomass production compared to the tidal freshwater forested wetlands [55], illustrating the importance of belowground productivity in maintaining wetland elevation [56,57], and highlighting the role of plant-mediated changes in ecological function. These results suggest that the conversion from freshwater forested wetland to oligohaline marsh will lead to more resilient wetland communities with a greater capacity to adjust to rising sea levels [58].

CO_2_-induced shifts in plant community composition can have an overwhelming impact on both wetland productivity [59] and decomposition [60], illustrating the important role of plant community dynamics in mediating future environmental conditions. Generally, decay rates were highest in the mudflat mesocosms compared to the vegetated mesocosms, which may be due to changes in nutrient availability and subsequent microbial activity [61] in the absence of vascular plants. This trend was moderated by elevated CO_2_ concentrations, which stimulated decay rates; however, this effect was only observed in the mixed community. Although CO_2_-induced changes in litter composition can influence decay rates [26,62], the use of a standardized carbon substrate in the current study isolated only those effects associated with changes in the hydro-edaphic environment and microbial community [63], both of which can be shaped by the functional traits of different plant communities as they respond to elevated CO_2_. For example, exposure to elevated CO_2_ can alter oxygen and carbon availability through increased root exudates [28] and root turnover [64], which can affect microbial activity and decay [65,66,67,68]. While root exudates were not directly measured, results from the current study provide evidence of a CO_2_ effect on labile carbon decay that is not related to changes in plant litter composition.

Direct stimulation of biomass production by elevated atmospheric CO_2_ has been well documented in coastal wetlands, especially those dominated by C_3_ species [69]. In the current study, we did not observe significant effects of elevated atmospheric CO_2_ on net annual above- or belowground biomass production in the C_3_-dominated *S. americanus* marsh or the mixed communities and only a minor effect on *N. biflora* aboveground production. While the lack of response by the C_3_-dominated communities was surprising, reports of CO_2_ enhancement of plant production are inconsistent throughout the literature [70]. Other studies have shown that the CO_2_-fertilization effect can vary depending on a multitude of factors including resource availability [71,72,73,74] and edaphic conditions [24,75,76]. For example, biomass of a C_3_ mangrove (*Avicennia germinans*) was increased by elevated CO_2_ treatment only when grown alone under high nitrogen availability [71]. In the current study, biomass production of *N. biflora* was stimulated by elevated CO_2_ treatments only when sediment was added to the mesocosms. Sediments deposited during storm events or restoration efforts have been shown to stimulate above- and belowground production in coastal wetlands by ameliorating flooding stress and nutrient deficiency [77,78,79]. Other work has found that CO_2_ enrichment effects on primary production typically occur when water use efficiency and nutrient availability are optimal [70]. The addition of sediment to mesocosms may have increased nutrient availability, which aided the response of *N. biflora* to CO_2_.

Landfalling hurricanes can deposit significant volumes of sediment in coastal wetlands [33,34]. In regions where natural tidal or riverine flooding is restricted, hurricanes and winter storms are a primary source of sediment [80] that can increase elevation capital and stimulate belowground production [36]. Both field and greenhouse experiments have shown that sediment subsidies can ameliorate the negative effects of sea-level rise by improving hydro-edaphic conditions that support greater plant productivity and wetland elevation gain over time [37,79,81,82]. However, sediment addition to mesocosms did not have a direct effect on belowground biomass production (Figure 5A), although sediment treatments did stimulate decay in the mudflat, likely through adding nutrient-rich sediments [61,81], and the greatest impact of sediment deposition was on elevation capital and subsequent elevation change trajectories (Figure 5C). As expected, sediment addition to mesocosms initially raised soil elevations, as occurs in the field, but subsequent rates of elevation gain were lower than in mesocosms without sediment. Similarly, in a manipulative field experiment that quantified the effect of sediment subsidies to wetland elevation change, the authors of [83] observed initial increases followed by a decline in wetland elevation that was associated with compaction of the underlying native soil. Over 2.5 years, wetlands treated with 2.3–20.3 cm of sediment subsided to pre-treatment elevations that were equivalent to the natural reference marshes. Similarly, compaction and decay may account for the slower elevation gains in mesocosms with added sediment.

## 4. Methods

### 4.1. Experimental Design

To investigate the effects of multiple drivers on ecosystem function along the coastal wetland landscape continuum, we simulated hurricane disturbance by adding sediment to four vegetation combinations in mesocosms (described below) exposed to ambient and elevated concentrations of CO_2_. The mesocosm study was conducted in the Wetland Elevated CO_2_ Experimental Facility at the U.S. Geological Survey (USGS), Wetland and Aquatic Research Center, in Lafayette, LA, USA. Treatments were applied using a split-plot with factorial subplots design consisting of (1) two CO_2_ treatments (ambient, ≈380 ppm; elevated, ≈720 ppm) applied at the whole-plot level, (2) four vegetation combinations (forest; marsh; forest/marsh mixture; mudflat) applied at the subplot level, and (3) two sediment treatments (sediment deposition; no sediment) applied at the subplot level. Each greenhouse (*n* = 4) contained two experimental units per community × sediment combination (*n* = 16) for a grand total of 64 mesocosms (Figure S1).

### 4.2. Mesocosm Design

Wetland community treatments (mesocosms) contained *Nyssa biflora* Walter seedlings, a C_3_ species, in the forest mesocosms; *Schoenoplectus americanus* (Pers.) Volkart ex Schinz and R. Keller, a C_3_ sedge, in the marsh mesocosms; and a combination of *N. biflora* and *S. americanus* in the mixed mesocosms. The fourth community, the mudflat, did not contain any vascular plants, and all mesocosms contained the same native soil from a *S. americanus* marsh. *Nyssa biflora* is a native tree species distributed throughout the Eastern United States, including the Atlantic and Gulf Coastal Plain, the Eastern Mountains and Piedmont, the Great Plains, and the Northcentral and Northeast regions [84]. *Schoenoplectus americanus* is a native sedge species distributed across North America from the Atlantic and Gulf Coastal Plain into the Arid West and into the western mountains, valleys, and coastal regions, as well as in the Caribbean [84]. Marsh sods dominated by *S. americanus* were collected from Big Branch National Wildlife Refuge, Louisiana, USA, in May 2012, before peak growing season, near the forest–marsh ecotone (Figure S2). Following collection, sods were cut to a depth of 20 cm and placed in 5-gallon buckets that contained a 5 cm thick bottom layer of pea gravel to improve drainage. To prepare the mesocosms for planting, all *S. americanus* vegetation was clipped to the soil surface. To ensure the complete elimination of *S. americanus* vegetation from the forest and mudflat mesocosms, those mesocosms were then flooded to a depth of five centimeters and re-clipped until no resprouting occurred (around two weeks). In the marsh and mixed mesocosms, flooding was not imposed, and *S. americanus* vegetation was allowed to re-grow. *Nyssa biflora* seeds were collected from a coastal freshwater forest in Georgetown, South Carolina, USA, and shipped to the USGS facility where they were germinated in commercial potting soil under ambient CO_2_ (non-enriched) conditions and allowed to grow for four months. Single *N. biflora* seedlings were transplanted to the forest and mixed mesocosms (Figure S1).

### 4.3. Experimental Conditions

Half the number of mesocosms (*n* = 32) were subjected to atmospheric CO_2_ concentrations of ≈380 ppm (ambient CO_2_ during study period, 2012–2014) and half to ≈720 ppm CO_2_ (elevated CO_2_). Ambient and elevated CO_2_ treatments were applied to the whole plot, i.e., the greenhouse (*n* = 2 per CO_2_ treatment) using an automated delivery system to ensure continuous targeted CO_2_ concentrations by adding industrial-grade CO_2_ (supplied by Airgas, Lafayette, LA, USA). The automated feedback system measured CO_2_ concentrations using a dual-beam, steady-state infrared gas analyzer (Gascard II, Edinburgh Instruments, Ltd., Livingston, UK) that regulated automated flow meters for each greenhouse (Cole-Parmer Instrument Company, Vernon Hills, IL, USA) (see [71] for details). Mesocosms were acclimated in ambient and elevated CO_2_ greenhouses, separately, for 10 weeks prior to sediment treatment.

Following the passage of Hurricane Isaac over the collection site (29 August 2012) [85], root-free and rhizome-free sediment was collected from the tidal creek adjacent to the marsh sod collection site and mixed with water to achieve a slurry of 70% water and 30% sediment by volume. To mimic hurricane sediment deposition, the sediment slurry was added to half the number of mesocosms (*n* = 32) to increase the soil surface by five centimeters (accounting for initial compaction after one week).

Mesocosms were flooded with freshwater to the soil surface and maintained at this depth throughout the study duration to mimic the relatively static flooding regime commonly observed in freshwater forests undergoing transition to oligohaline marsh in coastal Louisiana. To maintain plant vigor, a nutrient solution was applied to all mesocosms twice per month that provided mmol L^–1^ of N (0.005) as NH_4_Cl; P and K (0.00125) as KH_2_PO_4_; S (0.0025) as MgSO_4_; Ca (0.00625) as CaCl; Fe (0.00125) as FeEDTA solution; and micronutrients B, Cu, Mn, Mo, and Zn (0.00125) as H_3_BO_3_, CuSO_4_•5H_2_O, MnCl_2_•4H_2_O, H_2_MoO_4_•H_2_O, and ZnSO_4_•7H_2_O, respectively.

### 4.4. Data Collection

Data collection began in September 2012 and concluded in September 2014. Above- and belowground production was measured for herbaceous (*S. americanus*) and woody (*N. biflora*) species. *Schoenoplectus americanus* aboveground production was measured by harvesting all dead material from the mesocosms monthly and all live and dead material from a final harvest at the end of the study period [24]. Harvested aboveground material was dried to a constant mass at 60 °C and weighed, and the cumulative biomass produced over time was used to estimate a rate of aboveground primary production (g m^−2^ y^−1^). Litterfall from *N. biflora* seedlings was monitored weekly, and dead leaves were collected upon abscission. At the end of the two-year study, *N. biflora* saplings were harvested prior to seasonal leaf senescence and separated into components (leaf, stem, roots). All harvested material was dried to a constant mass at 60 °C and weighed. The cumulative biomass produced over time was used to estimate a rate of aboveground primary production (g m^−2^ y^−1^).

Belowground biomass production was estimated using ingrowth bags [86], which integrates the net production, turnover, and decomposition of roots and rhizomes over time. Root ingrowth was measured in two separate one-year deployments over the course of the study. In each annual deployment, one root ingrowth bag (5 × 20 cm), constructed of 2.5 mm plastic woven mesh and containing *Sphagnum* peat, was placed in a randomly selected quadrant of each mesocosm. The ingrowth bags remained in the soil for one year, after which they were harvested, and roots and rhizomes were separated from the sphagnum peat. Roots were not separated by species, and belowground biomass values are reported at the community level. Belowground biomass was dried to a constant mass at 60 °C and weighed, and the cumulative biomass produced over one year was used to estimate a rate of belowground primary production (g m^−2^ y^−1^). Although these methods may over- or underestimate above- and belowground production, they provide a relative measure of the response to treatments.

Belowground cellulose decay was measured using the cotton strip technique [87]. Unprimed heavy canvas (12 oz duck, style #548; Tara Materials, Inc., Lawrenceville, GA, USA) comprised of 100% cotton (98% holocellulose) was cut into 10 cm wide by 30 cm long strips and placed vertically into the soil to a depth of 20 cm in each mesocosm. Two cotton strips were installed in each mesocosm quarterly after all vegetation, elevation, and physicochemical measurements were made. One cotton strip, serving as a reference control, was removed immediately after installation, and the second cotton strip (test strip) remained in the mesocosms until the cotton strips decayed by at least 50% [88]. To ensure a minimum of 50% decay, cotton strips deployed at lower soil temperatures remained in the soil for longer (e.g., 21 days during the winter event) than cotton strips deployed at higher soil temperatures (e.g., 7 days during the summer event). The length of deployment was determined following the temperature × time relationship quantified by [88,89]. Upon retrieval, cotton strips were rinsed with deionized water, air-dried, and cut horizontally into 2 cm sub-strips that were measured for tensile strength with a tensiometer (Mecmesin model, Dillon Quality Plus, Inc., Camarillo, CA, USA). Cellulose decay was estimated as cotton strip tensile strength loss per day (CTSL d^−1^) calculated as

% CTSL d^−1^ = [(1 − T/R) × 100]/t, (1)

where T is the force (N) required to tear the test strips, R is the force (N) required to tear the reference strips, and t is time (days) in the soil.

Soil surface elevation was measured quarterly with a miniature surface elevation table mini-SET [24] designed after the rod-SET used in a field setting [90]. The mini-SET consisted of a removable measuring arm that was attached to the edge of the mesocosm in one of two positions. Fiberglass pins were lowered from the measuring arm to the soil surface. The change in distance from the arm to the soil surface over time corresponded to the change in surface elevation. Elevation change was expressed as the difference between base elevation measured at the initial sampling event and each subsequent sampling event. For each experimental unit (mesocosm), 11 pin-level surface elevation change values from two positions (*n* = 22), over a period of two years following sediment addition, were used to estimate a rate of surface elevation change.

### 4.5. Data Analysis

All analyses were carried out in R [91]. We used linear mixed effects models (R package “lmerTest” [92]) and type III analysis of variance (ANOVA) to quantify the relationship between species, community, CO_2_, and sediment deposition treatments and aboveground biomass production (Equation (2)) and the relationship between community, CO_2_, and sediment deposition treatments and each of the three response variables (i.e., belowground biomass production, decomposition, surface elevation change) (Equation (3)), where the mesocosm is the experimental unit for both models. For surface elevation change, we first conducted a linear regression to calculate the mesocosm-level rate of elevation change and then used the resulting slopes as a dependent variable in the linear mixed effects models and ANOVA. For post hoc analysis, we estimated marginal means (R package “emmeans” [93]), a variation of least square means. We used the Satterthwaite approximation for degrees of freedom in all analyses, and the Tukey method for *p*-value adjustment.

Aboveground biomass production ~ species × community × CO_2_ × sediment + (1|pot) + (1|GH), (2)

where species is the individual plant species (*N. biflora*, *S. americanus*), community is the plant community (forest; marsh; forest/marsh mix; mudflat), CO_2_ is the CO_2_ treatment (ambient; elevated), sediment is the sediment treatment (deposition; no deposition), and random factors are greenhouse (GH) and mesocosm (pot).

Response ~ community × CO_2_ × sediment + (1|GH), (3)

where Response is the dependent variable (belowground biomass production in g m^−2^ y^−1^; decomposition rate in % CTSL d^−1^; or slope of surface elevation change), community is the plant community (forest; marsh; forest/marsh mix; mudflat), CO_2_ is the CO_2_ treatment (ambient; elevated), sediment is the sediment treatment (deposition; no deposition), and GH is the greenhouse included as a random factor.

All data presented here is available in Stagg et al. (2022) [49].

## 5. Conclusions

As climate and land-use change cause vegetation shifts in coastal wetland plant communities, the conversion of freshwater forested wetland to oligohaline marsh can facilitate changes in biomass production, decomposition, and soil surface elevation that are primarily regulated by the dominant herbaceous marsh species *S. americanus*. When *S. americanus* was present, above- and belowground biomass production was highest, decomposition rates were lowest, and wetland elevation gain was greatest, and this pattern was maintained regardless of CO_2_ and sediment deposition treatments. Sediment deposition facilitated CO_2_-enhanced production of *N. biflora*, but tree production rates still did not surpass the marsh production rates. Sediment deposition initially increased elevation capital in all communities, but subsequent rates of elevation gain were lower than in mesocosms without added sediment. Together these results indicate that encroachment of oligohaline marshes into freshwater forested wetlands can enhance belowground biomass accumulation and resilience to sea-level rise (Figure S3), and these plant-mediated ecosystem services can be augmented by periodic sediment pulses from storms and restoration efforts. However, the persistence of a storm layer will depend on post-deposition erosion and bioturbation, as well as the initial thickness and texture of the sediment [36]. Thus, recurring sediment subsidies may be necessary to maintain these benefits over long-term periods of sea-level rise [94].

## Figures and Tables

**Table 1:** ANOVA summary table reporting F- and *p*-values for main effects and interactions for aboveground primary productivity. Significant values in bold. Significant levels: *** *p* < 0.001.

Treatment	Aboveground Biomass Production	
	F-Value	*p*-Value
**Species**	139.416	<0.0001 ***
**Community**	83.826	<0.0001 ***
**CO_2_**	1.216	0.276
**Sediment**	3.612	0.063
**Species × Community**	213.463	<0.0001 ***
**Species × CO_2_**	1.202	0.278
**Species × Sediment**	1.355	0.25
**Community × CO_2_**	1.107	0.355
**Community × Sediment**	0.736	0.536
**CO_2_ × Sediment**	2.196	0.145
**Species × Community × CO_2_**	1.156	0.336
**Species × Community × Sediment**	1.957	0.133
**Species × CO_2_ × Sediment**	3.365	0.073
**Community × CO_2 ×_ Sediment**	2.585	0.064
**Species × Community × CO_2_ × Sediment**	2.768	0.052

**Table 2:** ANOVA summary table reporting F- and *p*-values for main effects and interactions for each of the response variables, belowground primary production, decomposition, and surface elevation change. Significant values in bold. Significance levels: ** *p* < 0.01, *** *p* < 0.001.

Treatment	Belowground Biomass Production		Decomposition		Surface Elevation Change	
	F-Value	*p*-Value	F-Value	*p*-Value	F-Value	*p*-Value
**Community**	15.012	<0.0001 ***	96.465	<0.0001 ***	108.633	<0.0001 ***
**CO_2_**	1.445	0.232	0.276	0.652	5.433	0.145
**Sediment**	3.848	0.052	30.22	<0.0001 ***	12.505	0.001 **
**Community × CO_2_**	0.447	0.72	5.759	0.001 **	1.34	0.273
**Community × Sediment**	1.762	0.158	24.68	<0.0001 ***	0.787	0.507
**CO_2_ × Sediment**	1.295	0.257	0.267	0.605	0.986	0.326
**Community × CO_2_ × Sediment**	1.503	0.218	1.244	0.292	0.283	0.837

## Data Availability

All data are available in Stagg et al. (2022) [38].

## Supplementary Materials

The following supporting information can be downloaded at: https://www.mdpi.com/article/10.3390/plants11091259/s1, Figure S1: Experimental design and setup, and data collection timeline; Figure S2: Location of marsh sod collection. Marsh sods dominated by *Schoenoplectus americanus* were collected from Big Branch National Wildlife Refuge, Louisiana, USA in May 2012 before peak growing season, near the forest-marsh ecotone. Louisiana wetland data provided by USFWS National Wetlands Inventory [95]; Figure S3: Effect of landscape-scale transition from freshwater forested wetland to oligohaline marsh on belowground production (right y-axis) and surface elevation change (left y-axis).
