# Supplementary material for: Presence of the Herbaceous Marsh Species Schoenoplectus americanus Enhances Surface Elevation Gain in Transitional Coastal Wetland Communities Exposed to Elevated CO2 and Sediment Deposition Events
Source: Plants (Basel). 2022 May 6;11(9):1259. doi: 10.3390/plants11091259 (PMC9102553; doi:10.3390/plants11091259)
Supplement: Supplementary file 1 [file plants-11-01259-s001.zip › plants-1653938-supplementary.pdf]

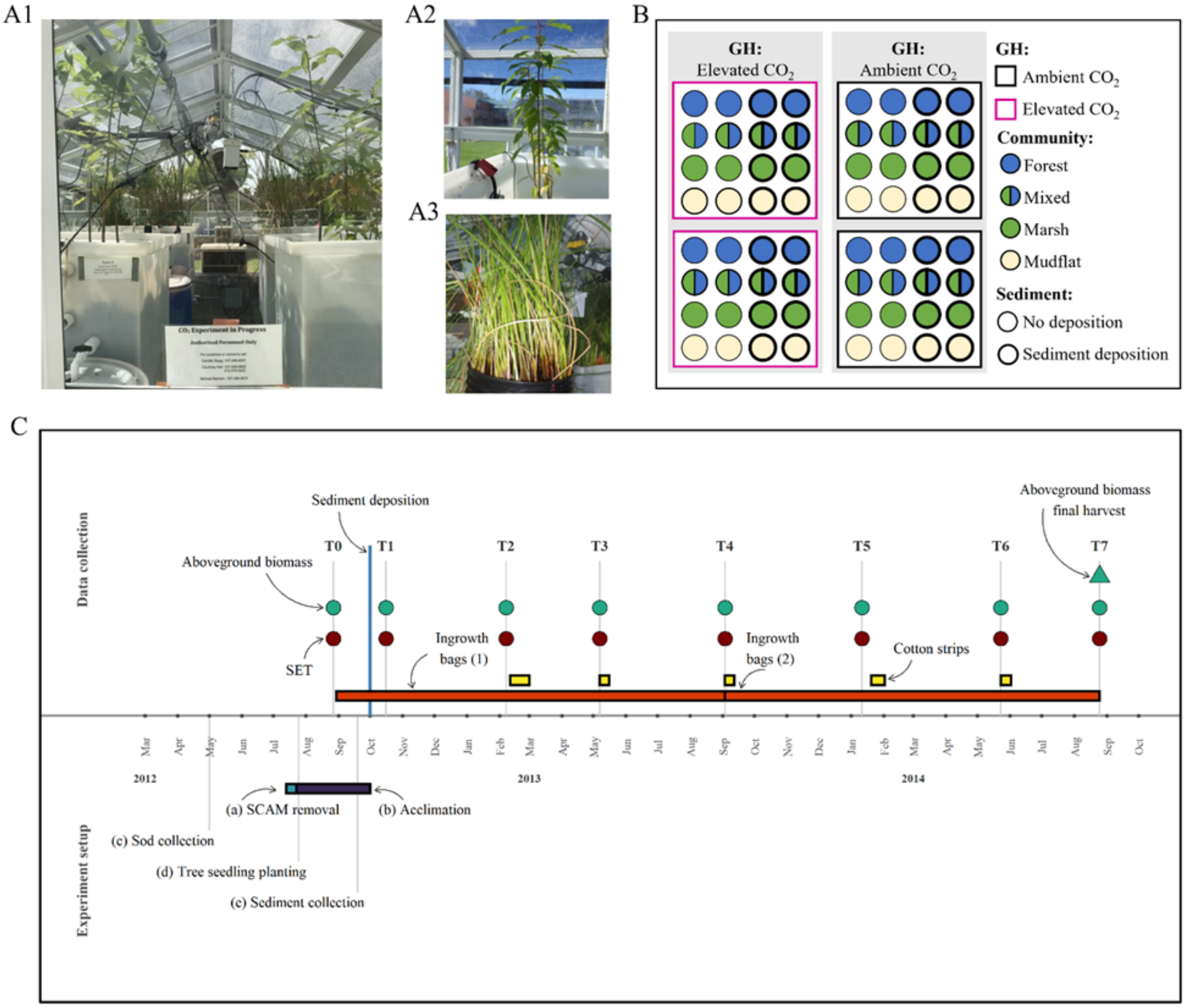

Figure S1. Experimental design and setup, and data collection timeline. A1) Mesocosms set up in tanks in greenhouse; A2) close-up of forest mesocosm (*Nyssa biflora*); A3) close-up of marsh mesocosm (*Schoenoplectus americanus*). B) Simplified schematic overview of experimental design-does not represent greenhouse position. C) Timeline detailing experimental setup and data collection events (a) To prepare the mesocosms for planting, all *S. americanus* vegetation was clipped to the soil surface. To ensure the complete elimination of *S. americanus* vegetation from the forest and mudflat mesocosms, those mesocosms were then flooded to a depth of five centimeters and re-clipped until no resprouting occurred (ca. two weeks). In the marsh and mixed mesocosms, flooding was not imposed and *S. americanus* vegetation was allowed to re-grow. (b) Mesocosms were allowed to acclimate for a ten-week period before sediment deposition treatments were applied. (c) All mesocosms contained the same native soil from a *S. americanus*-dominated marsh, collected from Big Branch National Wildlife Refuge, Louisiana, USA in May 2012. (d) Germinated *N. biflora* seedlings were transplanted to the forest and mixed mesocosms. (e) Root- and rhizome-free sediment was collected from the tidal creek adjacent to the marsh sod collection site following the passage of Hurricane Isaac and subsequently used for sediment deposition treatments in half of the mesocosms.

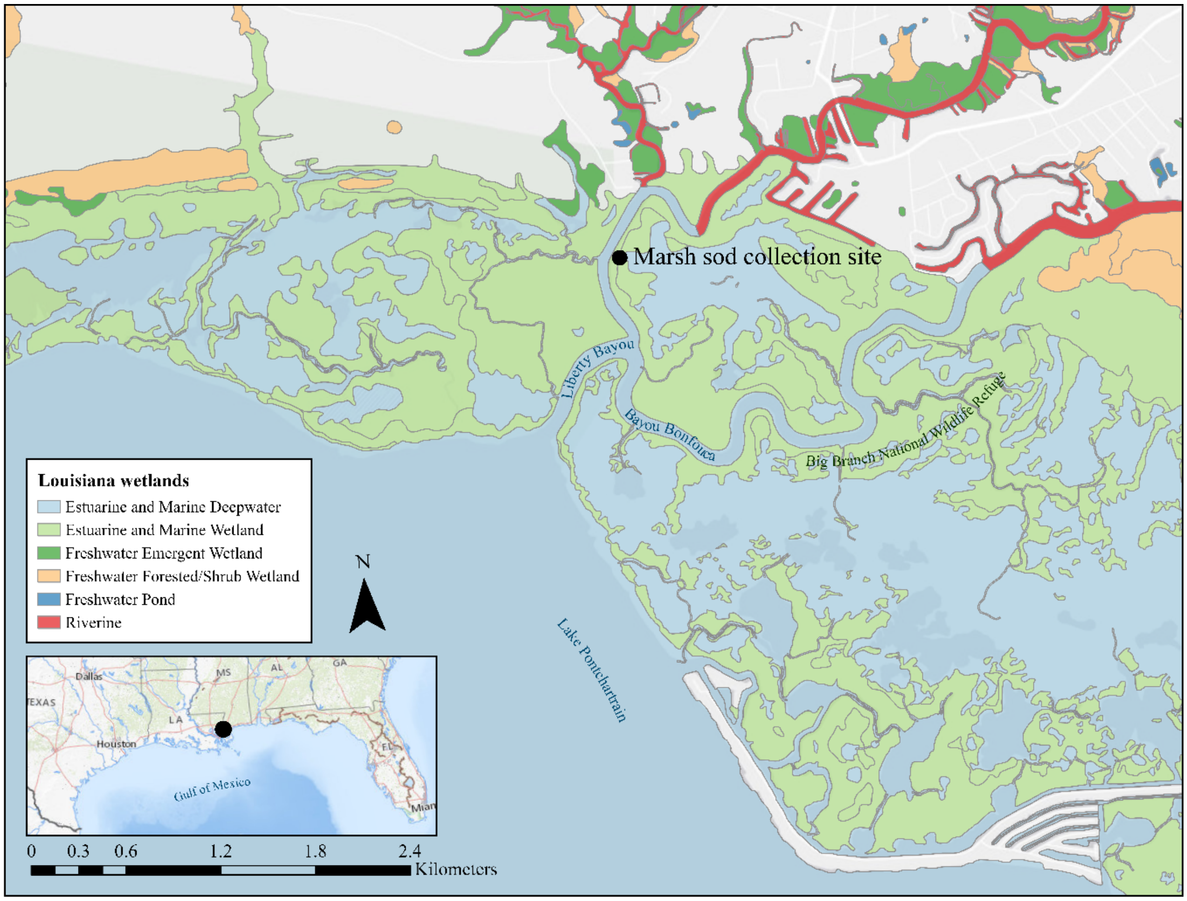

Figure S2. Location of marsh sod collection. Marsh sods dominated by *Schoenoplectus americanus* were collected from Big Branch National Wildlife Refuge, Louisiana, USA in May 2012 before peak growing season, near the forest-marsh ecotone. Louisiana wetland data provided by USFWS National Wetlands Inventory [1].

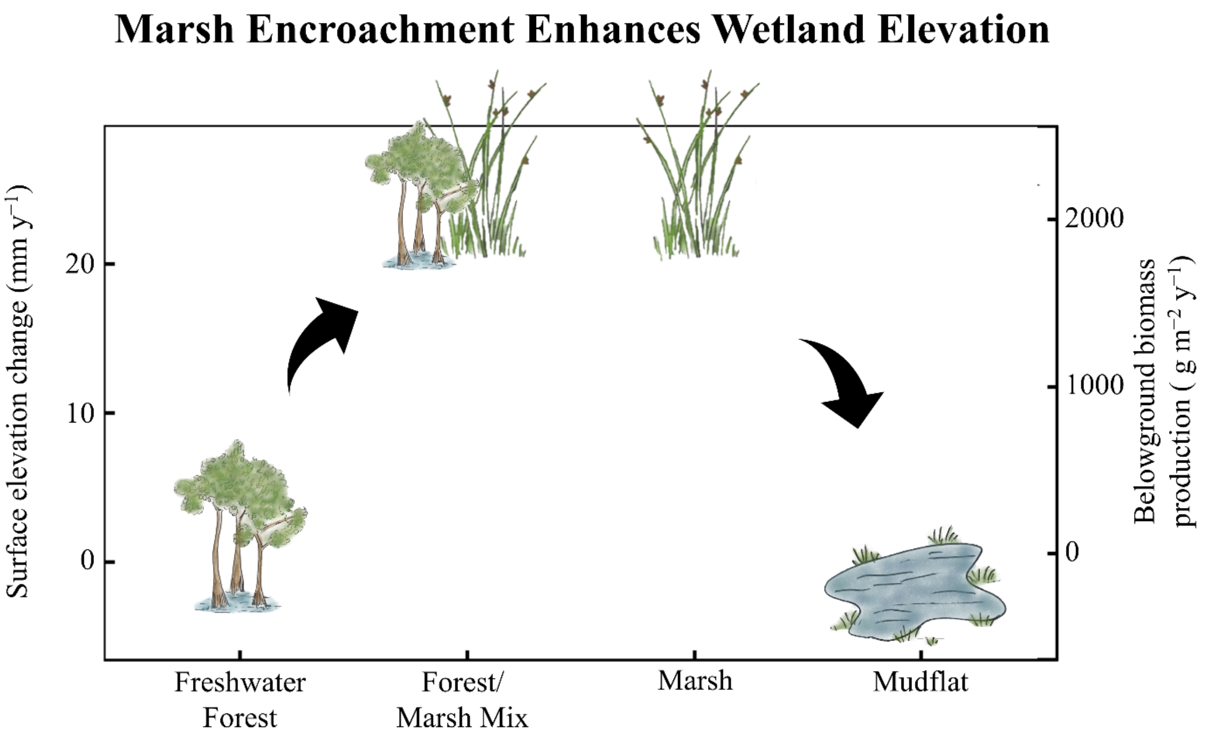

Figure S3. Effect of landscape-scale transition from freshwater forested wetland to oligohaline marsh on belowground production (right y-axis) and surface elevation change (left y-axis).

References

1. National Wetlands Inventory | U.S. Fish & Wildlife Service Available online: https://www.fws.gov/program/national-wetlands-inventory (accessed on 13 April 2022).
